# Improving the lives of millions through new double fortification of salt technology

**DOI:** 10.1111/mcn.12773

**Published:** 2019-05-31

**Authors:** Levente L. Diosady, M.G. Venkatesh Mannar, Kiruba Krishnaswamy

**Affiliations:** ^1^ Department of Chemical Engineering and Applied Chemistry University of Toronto Toronto Canada; ^2^ Department of Biomedical, Biological and Chemical Engineering & Food Science Program University of Missouri Columbia USA

**Keywords:** anemia, fortification, iodine, iron, maternal death, microencapsulation, salt

## Abstract

Micronutrient deficiencies (including iodine and iron deficiency) is a global health problem affecting one third of the world's population. Salt is an ideal carrier for food fortification as it is universally consumed at equal rates, independently of economic status, and it is industrially processed. Addressing iron and iodine deficiencies together is a challenge, due to interaction between iodine and iron, negating the effect of added iodine. This paper explains the development of an improved microencapsulation‐based technology to produce iron premix, which, when added to iodized salt, is stable and organoleptically indistinguishable. Ferrous fumarate was extruded, followed by cutting, sieving to achieve a size of 300–710 μm (salt grain size). Agglomerated extrudates were microencapsulated (5% hydroxypropyl methylcellulose and 5% soy stearin) to form iron premix. Microencapsulation ensures that the added micronutrients are stable without interaction or degradation. Double Fortified Salt is formed by blending iron premix with iodized salt (1:200 ratio). This technology was transferred to India for industrial scale‐up. The public distribution system was utilized to establish and monitor an efficient distribution network for DFS in a transparent manner. The scale‐up process was initially demonstrated in the state of Uttar Pradesh, following its success two more Indian states have started distribution of DFS. At present, the DFS with iron and iodine is reaching 60 million people in India. This important health intervention technology through food fortification has the potential to be scaled globally to ensure a world free from iron deficiency anemia.

**Key messages**

- Double Fortified Salt (DFS) is a cost‐effective and sustainable means to reduce iron deficiency anemia.
- Microencapsulated ferrous fumarate matches salt grains in size, colour, density and is not noticeable by the consumer when mixed with iodized salt.
- Local capacity building was used to produce DFS, with distribution through the Public Distribution Systems, targeting low income beneficiaries on a sustained basis.
- DFS currently reaches 60 million consumers in three Indian states (Uttar Pradesh, Madhya Pradesh, and Jharkhand)
- DFS technology developed is readily scalable for expanded coverage in India and other countries where iron deficiency is wide spread.

## 1 INTRODUCTION

Iron deficiency is a major cause of reduced immunity, reduced work capacity, maternal and infant mortality, impaired development in children. On the other hand, iodine deficiency is the largest cause of preventable mental and developmental problems (Diosady & Mannar, 2017). Iron deficiency anemia during pregnancy is a risk factor for preterm delivery, low birth weight and, in early life, can impair cognitive development (Allen, 2000; McLean, Cogswell, Egli, Wojdyla, & De Benoist, 2009). Nearly 1 billion people live in regions at risk of iodine deficiency, whereas iron deficiency affects 2 billion people, mostly in low income countries. In India, it affects more than 50% of women and 70% of children (Bentley & Griffiths, 2003; Toteja et al., 2006).

Dietary diversification, micronutrient supplementation, and food fortification are methods suggested by World Health Organization (WHO) to combat micronutrient deficiencies (Allen, Benoist, Dary, & Hurrell, 2006). Iron supplements are expensive and are not always affordable to populations with low incomes; also it is an active intervention that requires people to remember to take their supplements. Dietary diversification may not always be culturally appropriate or are too expensive (e.g., animal source). Food fortification is a cost effective, passive, and efficient intervention to combat micronutrient deficiencies.

Iron and iodine deficiencies can be addressed through large scale fortification of staple foods widely consumed in regular quantities independent of socio‐economic status (Allen et al., 2006; Diosady & Mannar, 2017). In many parts of the world, poor rural populations do not participate in the cash economy of manufactured foods and have limited access to processed staples such as cereal products, cooking oils, and dairy products. They do however purchase salt, which is typically centrally processed and purchased or bartered reaching even the poorest rural consumers (Allen et al., 2006; Diosady & Mannar, 2017).

Salt iodization is administered in more than 120 countries due to which majority of the world's population has regular access to iodized salt (WHO, 2014). This has resulted in a significant decrease in the prevalence of iodine deficiency diseases. It would therefore be prudent to use the existing iodization infrastructure to deliver iron along with iodine through salt to the deficient populations. Addressing iron deficiency through fortified salt has been proposed since the 1960s (Rao & Vijayasarathy, 1975). However, the reactivity and taste of most iron compounds were a challenge in developing a viable iron fortified salt. Iodine and iron interact, reducing the bioavailability of iron and leading to sublimation of iodine. Therefore, a technology that separates the micronutrients in salt and prevents interaction is required for double fortification of iron and iodine.

The challenges to salt double fortification hinge on the need to make fortification essentially transparent to the consumer. Thus, the Double Fortified Salt (DFS) should look, feel, and taste the same as iodized salt. Iodine, in the form of dissolved potassium iodate, is sprayed onto salt to form iodized salt. Iron has to be added at much higher levels compared with iodine (1,000 ppm vs. 30 ppm). The iron compound must be separated from the iodine (to prevent interactions)—therefore it must be added as solid particles. This requires matching the iron premix particle size to that of salt grains. Matching of the organoleptic characteristics of the premix to that of table salt involves selecting a bland tasting, well‐absorbed and inexpensive iron source. In this paper, we will explain the process development for new DFS from lab scale to large scale distribution capable of reaching millions of people in India.

## 2 METHODS

### 2.1 Process development

There are a number of technical issues that needs to be addressed in order to simultaneously fortify salt with iron and iodine. Iron compounds typically have a foul taste and, when oxidized to the ferric form, have limited bioavailability and unattractive, rust‐like colour (Diosady, Alberti, Ramcharan, & Mannar, 2002). Ferrous compounds react with potassium iodate to form elemental iodine, which readily sublimes, thus rendering salt iodization ineffective (Diosady, Alberti, Ramcharan, et al., 2002). Therefore, it is critical that the iodine and iron compounds be separated physically from each other in DFS to prevent their chemical interaction. Microencapsulation was selected as the technological means of salt double fortification (Diosady, Alberti, & Mannar, 2002). Although it would be simpler to encapsulate the relatively low quantities of added iodate, this is clearly impractical, due to the huge investment in place for spraying iodate on salt in salt iodization programs. Accordingly, we concentrated on encapsulating iron compounds.

Numerous iron forms were evaluated in an effort to optimize the cost and performance of the tested compounds. Ferrous fumarate was selected as the most appropriate iron source, as it is bland in taste, relatively inexpensive, and bioavailable. But, it has a dark red colour that needed to be masked before addition to table salt. Since it is impractical to admix very small particles to salt in a low resource setting, in initial work supported by the Micronutrient Initiative, Ottawa, Canada (Oshinowo, Diosady, Yusufali, Wesley, & Mannar, 2017), a laboratory technique was developed for agglomerating ferrous fumarate to approximate the size of salt grains using a pan coater. The ferrous fumarate was first agglomerated using hydroxypropyl methyl cellulose, then coated with titanium dioxide to mask the red colour of the ferrous fumarate. The particles were overcoated with soy stearin (SS)—fully hydrogenated soy oil—which is readily digested and melts less than 70°C. This is important, as 500 μm premix particles would a have a grainy, sand‐like mouthfeel, unless the iron is released from the microcapsules during food preparation (Oshinowo, Diosady, Yusufali, & Wesley, 2012). The iron premix was diluted to a ratio of 1:150 or 200 with salt to produce DFS. DFS made with this approach was stable at elevated temperature and humidity for more than 6 months. Consumer acceptability was demonstrated in five countries in Africa and Asia and developed quality assurance and control techniques to ensure product efficacy and safety (Oshinowo, Diosady, Yusufali, & Laleye, 2004). In field tests using the normal distribution system in Nigeria and Kenya, the DFS retained practically all its iodine content over several months (Oshinowo, Diosady, Yusufali, & Wesley, 2007).

The premix production process was scaled up, initially by using Wurster type fluidized bed agglomerator coater, from the laboratory to 5, 25, 60, 300, and finally 600 kg batches. This technology was transferred to a pharmaceutical manufacturer in India. DFS using this premix was stable at elevated temperature for more than a year (Oshinowo et al., 2012; Li, Diosady, & Wesley, 2010). It was used in preparing school lunches for 3.4 million children in Tamil Nadu state in India. The program has since been expanded to 5.5 million children across the state now fully supported by the state government.

**Figure 1:**
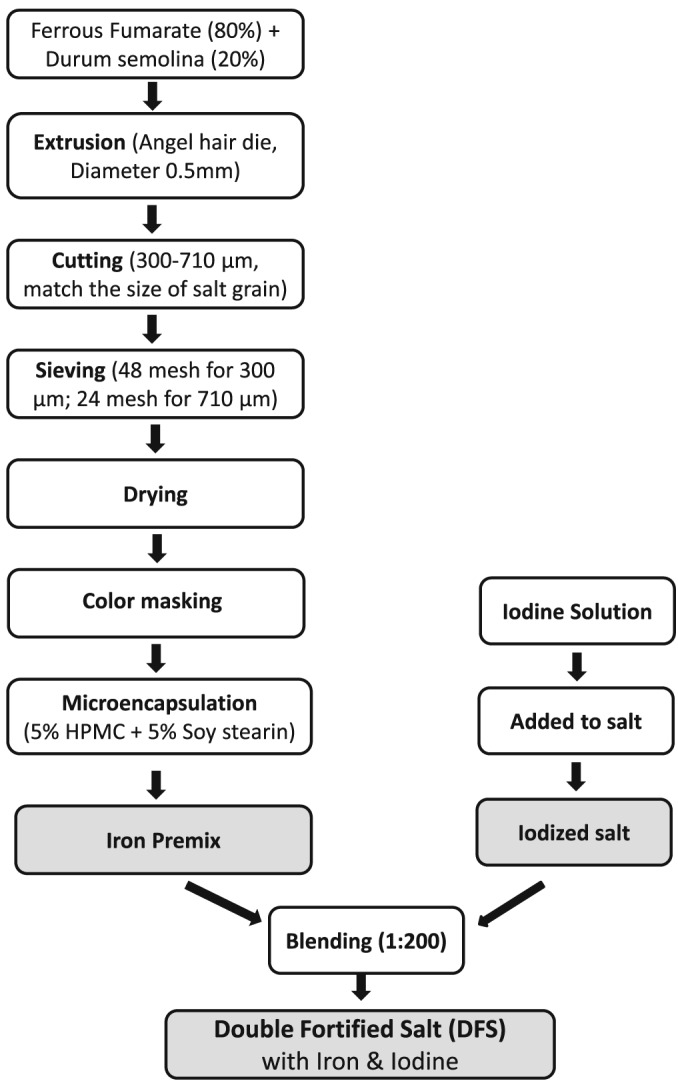
Process flow diagram for Double Fortified Salt technology

However, the fluidized bed technology is expensive and produced iron premix particles that had a low density, which resulted in the premix floating on water when salt was washed (Oshinowo et al., 2012). Accordingly, another approach to iron premix manufacturing was developed. The technology consists of preparation of a ferrous fumarate dough with an edible flour, water, and vegetable oil and extruding it through a fine, 0.4 mm‐sized angel‐hair pasta dye, using a restaurant‐scale pasta extruder, processing typically up to 1 kg/h. The strands of ferrous fumarate are cut to an equivalent size, to form cylinders ~500 μm in diameter. The particles were then coated with titanium dioxide in a pan coater or a Glatt Air Systems laboratory fluidized bed coater, to provide a bright white surface and microencapsulated by spraying them with SS (a hydrogenated soy oil) to provide a robust waterproof encapsulation (Li, Yadava, Lo, Diosady, & Wesley, 2011). During the field testing of previous version of DFS formulation in India, it was observed that the iron premix containing SS coat tends to float in water. While cooking with DFS, consumers may remove the floating premix from cooking water assuming it to be impurities (Yadava, 2008). This is a major problem to be addressed as consumers might not consume adequate amounts of iron from previous version of DFS. This led to developing the new DFS formulation. Extrusion agglomeration, colour masking, and microencapsulation coating are the three main process in iron premix production (Yadava, 2008). Hydroxypropyl methyl cellulose was added along with SS during the microencapsulation step. This new iron premix was then added to iodized salt at 1:200 ratio to form improved DFS. Figure 1 illustrates the process flow diagram for production of DFS.

**Figure 2:**
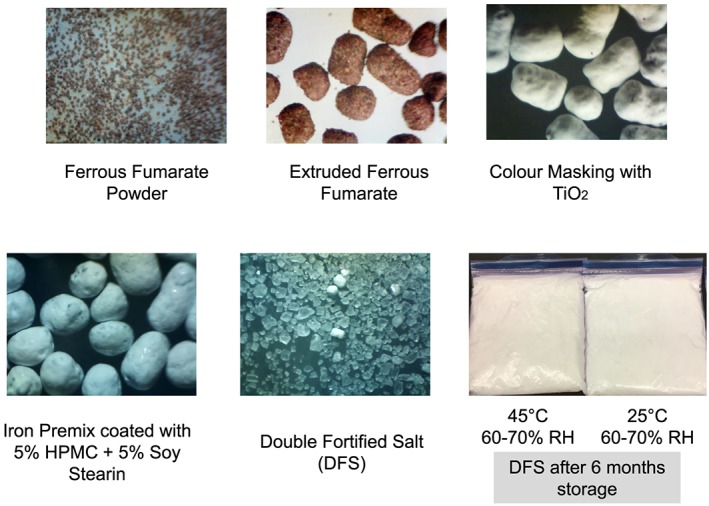
Product development stages in Double Fortified Salt

This process was readily scaled up to pilot scale, using a continuous forming extruder with a capacity of approximately 25 kg/h at JVS Foods Ltd. in Jaipur, India. The strands of extruded ferrous fumarate were spherolized to form nearly spherical particles of ~500 μm in diameter and then coated with 30% titanium dioxide in a candy coater, followed by 5% to 10% of hydroxypropyl methyl cellulose (HPMC) overcoated with 5% to 10% SS. This process flow can be visualized in Figure 2. Image showing step by step DFS product development. Material from this pilot scale process was tested for stability at 25°, 35°, and 45°C for up to 2 years, to ensure that iodine was retained, and the iron remained in its ferrous form.

### 2.2 Iodine stability test

In DFS, iron is microencapsulated with coating materials to prevent an interaction between iron and iodine. Measuring iodine stability over periods of time provides information about the encapsulation of iron indirectly. If iron is not properly encapsulated, exposed Fe^2+^ will react with iodate, which will lead to displacement of iodine from the iodate, and iodine is lost by sublimation process. The stability of iodine in salt fortified with the iron premix from both pilot production in India and the lab were investigated. Iodometric titration was used to determine the stability and retention of iodine in DFS at three different storage temperatures (25°C, 35°C, and 45°C) for 0 week, 2 weeks, 1, 2, 4, and 6 months.

### 2.3 Iron analysis

Inductively Coupled Plasma‐Atomic Emission Spectrometry (ICP‐AES) was used to analyse the iron content in DFS samples. The salt samples (1.0 g) were digested using 10 ml concentrated HNO_3_ in a 25‐ml volumetric flask on a hot plate for 20 min. The digested sample was made up to 25 ml using deionized water. For ICP‐AES analysis, 1 ml of the digested sample solution was mixed with 9 ml 5% HN0_3_ in 15 ml centrifuge tubes. The samples (*n* = 4) were analysed for elemental iron at 259.939 nm wavelength. PlasmaCAL Multi‐Element Standards (Q.C. Std. 4) were used for calibration and the quantifiable detection limits (axial) for the analysed elements were between 0.01 to 0.005 ug/ml. The iron concentration in the digested salt sample was calculated using the following equation: (*W* = (*ρ* × dilution factor × *V*)/*m*); where, *W* is the mass fraction of the element in the solid sample (mg/g); *ρ* is the concentration of the element in the sample (mg/L) obtained from ICP‐AES; *V* is volume of the digested sample (L); *m* is the mass of the digested sample (g).

## 3 RESULTS AND DISCUSSION

**Figure 3:**
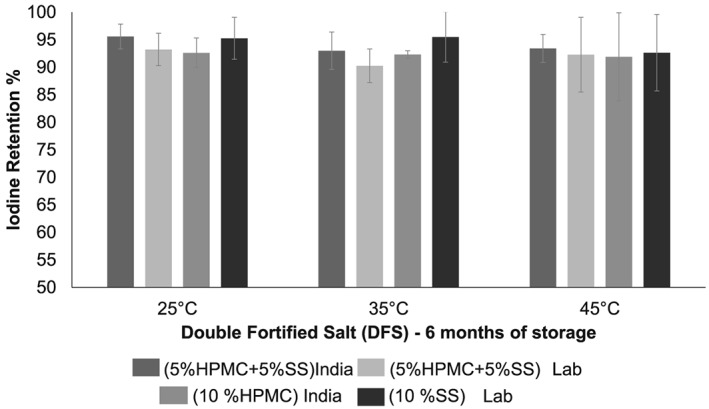
Iodine retention (%) in Double Fortified Salt samples stored at 25°C at RT, 35°C at 60–70% RH, 45°C at 60–70% RH after 6 months(Footnote: (a) India 2015 pilot batch coated with 10% hydroxypropyl methyl cellulose (HPMC); (b) lab 2016 batch coated with 10% soy stearin; (c) lab 2016 batch coated with 5% HPMC and 5% SS; (d) India 2016 batch coated with 5% HPMC and 5% SS)

The results for DFS iodine stability after 6 months are presented in the Figure 3. The DFS was produced by mixing iron premix with salt sprayed with 2% iodine. Four premix combinations were studied: (a) India 2015 pilot batch coated with 10% HPMC; (b) lab batch 2016 coated with 10% SS; (c) lab batch 2016 coated with 5% HPMC and 5% SS; (d) India 2016 batch coated with 5% HPMC and 5% SS. About 90–95% iodine retention was observed for all DFS samples after 6 months of storage clearly seen from Figure 3. Premix sample coated with (5% HPMC + 5% SS) from 2016 India pilot scale study proved to be the best combination based on iodine retention at three different temperatures. Premix sample 5% HPMC + 5% SS was prepared in the lab using a fluidized bed coating method. Due to differences in the size of the iron premix particles, the HPMC coat was not uniform leading to lower iodine retention compared with its Indian counterpart consisting of the same 5% HPMC + 5% SS combination. The colour and appearance of DFS samples were monitored at room temperature of 25°C, 35°C, 45°C at 60–70% relative humidity (RH). Salt samples containing India premix (5% HPMC + 5% SS) when compared were similar in appearance to table salt after 6 months of storage (see Figure 2). Comparing iodized salt and DFS, the ICP‐AES elemental profile was similar, with the DFS sample containing 10.36 ± 1.49 mg of Fe/10 g of salt after 1 year of storage at 45°C, 60–70% RH.

### 3.1 Efficacy

Efficacy studies using DFS conducted in 2008 showed 66% reduction in anemia and improvement in iron indicators, over 10 months (Andersson et al., 2008; Mannar, 2018). Studies conducted in Bangladesh and Sri Lanka for consumer acceptance of DFS in 2012, found good acceptance (Micronutrient Initiative ‐ Lanka Market Research, 2012; Mannar, 2018). A randomized controlled trial using DFS showed significant improvements in haemoglobin (+2.4 g/L), ferritin (+0.13 log10 μg/L), and body iron (+1.43 mg/kg), when compared with controls in all biomarkers of iron status, anaemic (haemoglobin <120 g/L), iron deficient (serum ferritin <12 μg/L) in female tea pickers in Darjeeling, India (Haas et al., 2014; Venkatramanan et al., 2017). The Darjeeling study, demonstrated that recipients of DFS compared to women tea pickers of reproductive age in India, had significant improvement in cognitive skills: perceptual, attentional, and mnemonic performance (Wenger et al., 2017; Mannar, 2018). It is true, however, that the effectiveness of DFS depends upon the concurrent causes of anemia, and how close are people to the anemia border line. National Institute of Health (2018), recommends is 18 mg/day for women of reproductive age, DFS provides approximately 30–50% of the daily recommended intake of iron, 10 mg/day from salt. The key advantage of the new DFS formulation lies in the fact that is almost identical in taste, colour, and odour to regular salt (Mannar, 2018). Though the microencapsulated ferrous fumarate does not deteriorate with time, it could be slightly observed in salt matrix (see Figure 2).

**Figure 4:**
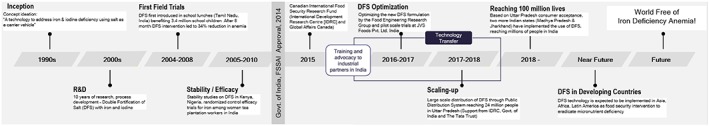
Journey of Double Fortified Salt: timeline

Over two decades of research and development from the inception of the idea to fortifying salt with iron, to contributions by researchers (Diosady, Alberti, & Mannar, 2002; Diosady, Alberti, Ramcharan, et al., 2002; Li et al., 2011; Oshinowo et al., 2004; Oshinowo et al., 2007; Oshinowo et al., 2012; Yuen, Li, Ue, Wesley, & Diosady, 2008) forms the basis of the new improved DFS formulation. Figure 4 depicts the timeline of DFS development. After a waiting period of several years, a breakthrough was achieved when the Food Standards and Safety Authority of India issued a final Gazette Notification on December 5, 2014, approving the revision of the standards for DFS, and include the new DFS formulation effective immediately (Ministry of Women and Child Development, Govt. of India).

### 3.2 Scaling up DFS

The following analyses for DFS have been completed to date, as prerequisites for scale‐up: efficacy, organoleptic (taste/colour/odour) properties, design and fabrication of process equipment, and other technical support. The next step involves evaluating the effectiveness of intervention through a targeted public distribution program, lay the foundation to launch through commercial channels, and to facilitate state and nationwide scale‐up (Mannar, 2018).

On the basis of our experience with salt production and marketing in India, we hypothesize that two distribution channels hold promise—(a) to target mid and lower income quintiles, a midtier market‐based channel, where salt is currently unbranded or locally branded is used. This salt is relatively moisture free and thus suitable for fortification and (b) to target the bottom income quintile, public distribution channels (eg. fair price shops). Also, mandating the use of fortification (over 100 countries require iodization of salt, and at least 50 require iron fortification). We recommend a phased approach to introduce the new DFS, first through targeted public distribution and market‐channel programs, then reaching the entire population by making DFS mandatory (Mannar, 2018).

### 3.3 Targeting DFS to the malnourished

The use of DFS has been not only been encouraged but also **mandated** in the *Mid‐Day Meal program* in Government of India run schools as well as the *Integrated Child Development Services* systems to support preschool children and pregnant/lactating mothers, as per a formal circular by the ministry in charge of those systems (Ministry of Women and Child Development Government of India, No. 5–4/2011, ND/Tech, 2011), although that use has not yet been implemented, largely because of the previous unavailability of an effective formulation. The Mid‐Day Meals program, reaches approximately 100 million school children across India everyday. In Tamil Nadu, the Mid‐Day Meal program has already distributed DFS to over 5 million school children in 33,000 schools, each school day for approximately 7 years.

Even larger than these two systems (in terms of reach) is the public distribution system in India, which targets the poor and reaches over 200 million people with its network of 500,000 Fair Price Shops. The public distribution system also reaches entire families, for potentially all meals and operationally builds on the distribution of packages of commodities to the home. The public distribution system was established by the Government of India under the Ministry of Consumer Affairs, Food, and Public Distribution and is managed jointly with state governments. A majority (an estimated nearly 80%) of the people in India who are currently covered under the public distribution system suffer from significant micronutrient deficiencies. However, less than 5% of foods provided through the public distribution system, are fortified with micronutrients. This is both a problem and an immense opportunity to help hundreds of millions of undernourished people. The public distribution system in many states already distributes iodized salt, which will pave the way for the introduction of DFS using the same procurement and distribution infrastructure.

### 3.4 Technology transfer and scale‐up

**Figure 5:**
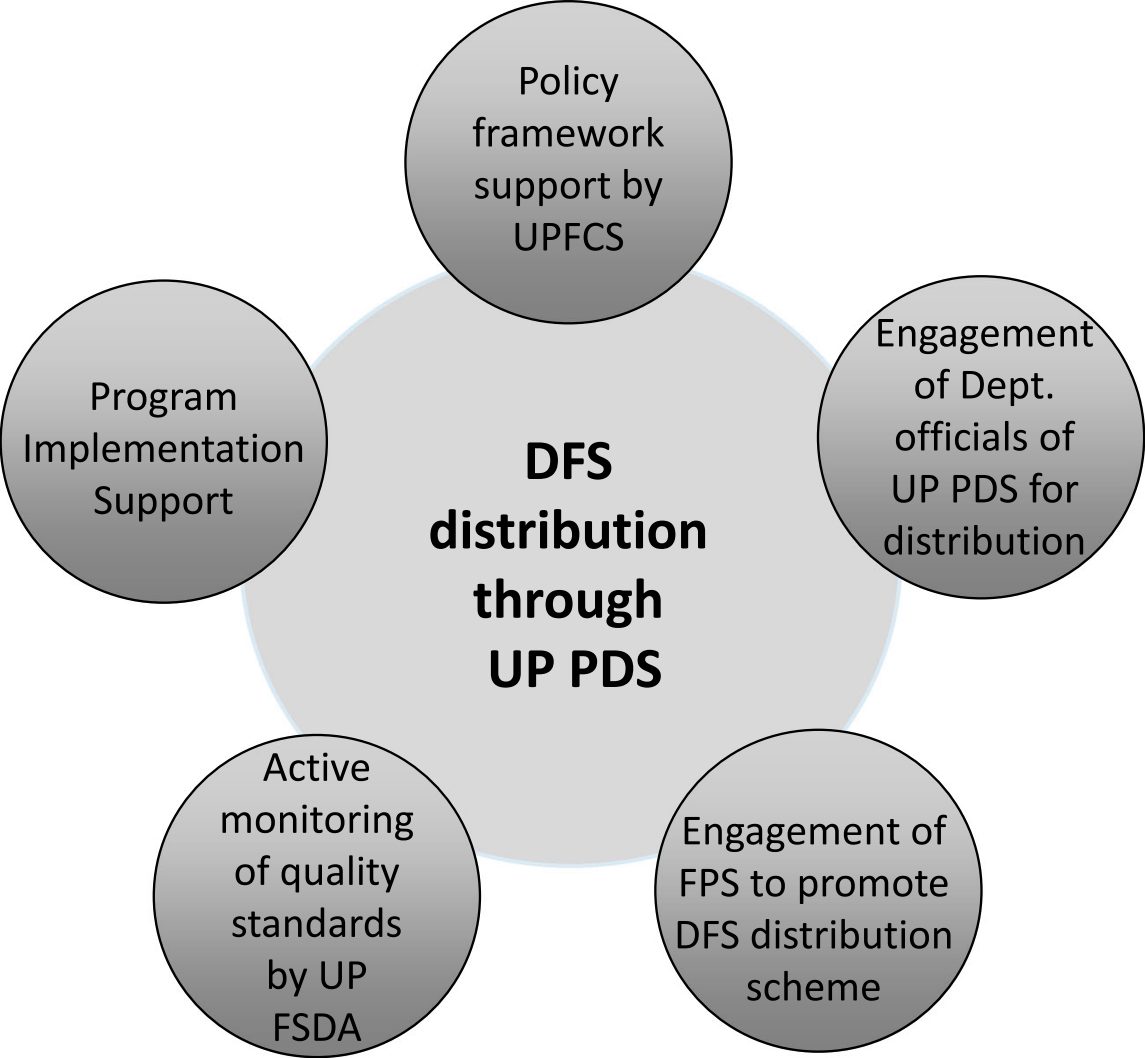
Double Fortified Salt program implementation strategy framework in Uttar Pradesh, India(Footnote: UP: Uttar Pradesh, India; PDS: public distribution system; FPS: Fair Price Shops; UPFCS: Uttar Pradesh Food & Civil Supplies Department; FSDA: Food Safety and Drug Administration]

**Figure 6:**
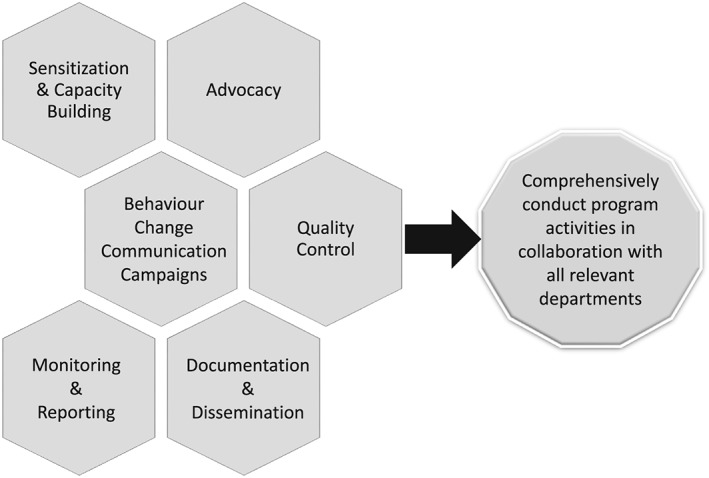
Double Fortified Salt program activities for scale‐up

In 2015, to facilitate the technology transfer and technical support for iron premix production (an industrial plant for producing the iron premix at scale was built and commissioned by JVS Foods of Jaipur), DFS production (technical assistance was provided to multiple salt processors to blend the iron premix with refined table salt), consumer research, and development of communication strategies. In parallel, with IDRC support for advocacy, communications, training, and monitoring of programs combined with an initial assessment of health impact, the Tata Trusts helped the Government of the State of Uttar Pradesh to establish a distribution network for DFS (to reach 24 million people in the State). A snapshot of program activities to implement the DFS scale‐up initiative is illustrated in Figure 5, to ensure smooth execution of the program yielding desired on‐ground results and meeting the stated objective of reaching the lower economic quantile in Uttar Pradesh State. The initiative is being implemented through an extensive network of fair price shops across Uttar Pradesh State that sells the salt to the beneficiaries at a subsidized price. Distribution through the public distribution system vis‐à‐vis open market will also help self‐select the target population, namely, the impoverished and help achieve better health status at scale. Figure 6 shows the key framework for effective DFS implementation strategy in Uttar Pradesh. Figure explains the need to coordinate within and among different groups, such as advocacy, communication campaigns for behaviour change, capacity building, quality control, monitoring, reporting, documentation, and dissemination of results to comprehensively conduct DFS program activities in collaboration with different departments.

Various outcomes and impacts of the project are being measured—uptake and consumption of the DFS, quality in terms of purity, iron, and iodine content and consumer acceptance of the salt. The program is also being evaluated for effectiveness.

The additional outcomes to be realized through implementation of the program in Uttar Pradesh include:

1. Local capacity to produce adequate quantities of quality DFS.
2. Availability of DFS for supply through public distribution system programs.
3. Regular availability of DFS at Fair Price Shops.
4. Improve knowledge of importance of DFS and willingness to pay.

The Bill and Melinda Gates Foundation is concurrently enabling baseline and end line surveys to evaluate the effectiveness of the program based on coverage, utilization of DFS by the target population, impact on anemia and iron status. More recently, two more Indian states (Jharkhand and Madhya Pradesh) have followed the Uttar Pradesh model and started similar programs to distribute DFS and currently reaching 60 million people. This important health intervention technology can be readily scaled globally similar to Universal Salt Iodization Program.

## 4 CONCLUSION

The DFS with iron and iodine could be replicated across the world and potentially reduce all forms of iron deficiency by 50% and sustain the elimination of iodine deficiency. DFS is now distributed with state aid in three Indian states. The actual cost of the intervention is ~2.5 IR/kg, or 3US¢/kg salt, adding 10–20% to the cost of salt. In other terms, the cost is now approaching 12¢per person per year. This is sustainable under three circumstances—state support, mandated addition, and strong marketing. The 2008 and 2012 Copenhagen Consensus of eminent economists ranked micronutrient programs as the single most cost‐effective development intervention to enhance human welfare. Thus, DFS is economical and sustainable. DFS technology is ready to be introduced on large scale in other countries, as it builds on the infrastructure that has already been established by the salt industry over the past two decades to achieve universal iodization. Beyond iodine and iron, there is also potential for including additional micronutrients like (folic acid, vitamin B12, vitamin B1, zinc) in salt.

## CONFLICTS OF INTEREST

The authors declare that they have no conflicts of interest.

## CONTRIBUTIONS

LL Diosady is the PI for this project. His role is the technical direction of the program.

MG Venkatesh Mannar coordinates all aspects of the work in India.

K Krishnaswamy directed the laboratory work and technology transfer from University of Toronto.
